# Supplementary material for: Skin Phototype Could Be a Risk Factor for Multiple Sclerosis
Source: J Clin Med. 2020 Jul 26;9(8):2384. doi: 10.3390/jcm9082384 (PMC7464407; doi:10.3390/jcm9082384)
Supplement: Supplementary file 1 [file jcm-09-02384-s001.pdf]

Table S1: Demographic and MS clinical variables

| | **Patients (n=149)** | **Controls (n=147)** | **p value** |
|---|---|---|---|
| Age (years) | 41.4±10.5 | 41.3±12.5 | n.s. |
| Sex(%female) | 61.2% (n=90) | 71.8% (n=107) | n.s. |
| Onset age | 29.0±10.0 | | |
| Diseases duration | 11.5±9.0 | | |
